# Accessible, fast and easy fabrication of hydrophilic-in-hydrophobic microdroplet arrays

**DOI:** 10.1371/journal.pone.0263282

**Published:** 2022-02-25

**Authors:** Arianna Toppi, Martin Dufva

**Affiliations:** Department of Health Technology, Technical University of Denmark, Lyngby, Denmark; Texas A&M University College Station, UNITED STATES

## Abstract

Microdroplet arrays (MDAs) are powerful tools for digital immunoassays, high-throughput screening and single cell analysis. However, MDAs are usually produced with cleanroom processes, which are associated with high costs and low availability. Furthermore, in order to obtain robust and stable MDAs based on hydrophilic spots surrounded by a hydrophobic background, the chemistry must be strictly controlled, which is challenging using shared equipment. Here, we developed a new method to fabricate MDA substrates independently from the cleanroom. A small and low-cost in-house built system to collimate the light source was assembled for photopatterning a negative resist, and spots with diameters down to 4 μm were obtained, with only 3% to 5% spot-to-spot variation across the same sample and high batch-to-batch reproducibility. The use of a negative photoresist enabled the formation of a hydrophobic coating in solution which yielded high-quality MDAs. The feasibility for carrying out digital assays was demonstrated by measuring anti-Tau antibody in sample buffers containing bovine serum albumin, with no noticeable surface fouling. The reported, robust, cost-effective, and fast process could hence lower the threshold to fabricate and use MDAs for digital immunoassays and other microcompartmentalization-based applications.

## 1. Introduction

Microdroplet arrays (MDAs) are essential tools developed for partitioning dilute samples in ultra-small volumes, thereby enhancing the sensitivity of biological assays down to the single molecule level. Since sample micro-compartmentalization was described by Rotman in 1961 [1] for the study of single enzyme molecules activity, this method has been further developed and widely applied for the ultrasensitive detection and quantification of nucleic acids and proteins [2–4], as well as for the analysis of single cells [5, 6] and exosomes [7]. MDAs enable the parallel monitoring of thousands of reactions and biological processes, which makes them suitable platforms for high throughput screening and multiplexing [8–10]. One of the most common applications of the MDAs is the so-called digital ELISA, first described by Rissin et al. in 2010, which is based on the same principle as standard ELISA but the single enzyme-labelled immunocomplexes are isolated in small compartments in the presence of a fluorogenic enzyme substrate [11]. Consequently, a strong fluorescence signal is generated in each of the compartments containing the enzyme-labelled single analyte. From the number of positive and negative partitions, it is possible to determine the sample concentration; hence the name of “digital counting”.

Several approaches to fabricate substrates for sample micro-compartmentalization have been investigated. The majority of them requires the use of cleanroom (CR) facilities, which are costly and not widely available. Therefore, the application of CR-based processes can hinder the large-scale usage of the MDA technology. Examples of substrates exploited for sample partitioning are PDMS gaskets containing microcavities [3, 12–15], microwell arrays hot-embossed on cyclic-olefin co-polymer (COC) substrates [16] or bundles of optical fibres where microwells are produced on their tips by etching the fibre cores [11, 17, 18].

Another approach for fabricating MDAs is based on the use of biphilic surfaces, where patterns of hydrophilic areas are surrounded by a hydrophobic coating, and droplets are spontaneously formed by contact with an aqueous phase due to the difference in surface energies of the two surface materials. Hydrophilic-in-hydrophobic (HiH) layouts can be fabricated by etching fluorocarbon-based coatings on glass substrates, as described by Sakakihara et al. [19] and by Leirs et al. [20], who used CYTOP^®^ and Teflon-AF^™^, respectively. CYTOP and Teflon-AF are costly and require complicated fabrication processes (i.e., a patterned masking layer and subsequent reactive ion etching). An alternative method, previously described in our group, exploits a hydrophobic organosilane deposited as a self-assembled monolayer from a gas phase on a masked glass surface [21]. The masking layer is patterned using standard UV photolithography processes, and successively lifted-off to expose the hydrophilic areas. The major drawback of this CR-based method is its dependency on shared equipment in the CR which introduces risks of contaminations. Especially in academic research settings, it is not common to have a dedicated fabrication line, free of possible contaminations deriving from different processes and, in our experience, the use of shared equipment often resulted in frequent batch-to-batch variations. Another drawback of a CR is that not all labs have access to it, and it is costly to use. Therefore, since the quality of the HiH-MDAs is highly correlated with the robustness of the surface chemistry, various methodologies were explored to move the entire process out of the CR and have full control on the chemical conditions during fabrication.

We here describe a process based on the use of AZ^®^ nLOF 2020 photoresist as sacrificial layer for the formation of a hydrophobic coating made of Trichloro(1H,1H,2H,2H-perfluorooctyl)silane (PFOCTS) that enables rapid, low-cost, and robust fabrication of HiH-MDAs in a regular wet chemistry lab with only minor instrument investments.

## 2. Materials and methods

### 2.1. Materials

2” borosilicate glass wafers were purchased from Siegert Wafers GmbH, Germany. Photoresist AZ^®^ nLOF 2020 and AZ 726 MIF Developer were purchased from Micro-chemicals GmbH, Germany. Trichloro(1H,1H,2H,2H-perfluorooctyl)silane (PFOCTS), N-Methyl-2-pyrrolidone (NMP), (3-Glycidyloxypropyl)trimethoxysilane (GLYMO), AmpliFlu Red^™^ (AFR) were purchased from Sigma Aldrich, MS, USA. 3M^™^ Novec^™^ 7200 Engineered Fluid (Novec 7200) was purchased from Walbom A/S, Denmark. Anti-CD63 antibody [TS63] (Cat. No. ab59479) and FITC Conjugation Kit (Fast)—Lightning-Link^®^ (Cat. No. ab188285) were purchased from Abcam plc, UK. Horseradish peroxidase (HRP) conjugated anti-Tau 419–433 Antibody and Recombinant Human Tau-441 (2N4R) were purchased from Biolegend, CA, USA. Buffer solution (10 mM PBS, 136 mM NaCl, 1% (w/v) BSA, 0.1% (v/v) Tween-20, 0.36% (v/v) Bronidox, pH 7.4) used for protein and antibody dilutions was kindly provided by Nordic Bioscience, Denmark.

### 2.2. Fabrication of the hydrophilic-in-hydrophobic spot arrays outside the CR

**Fig 1:**
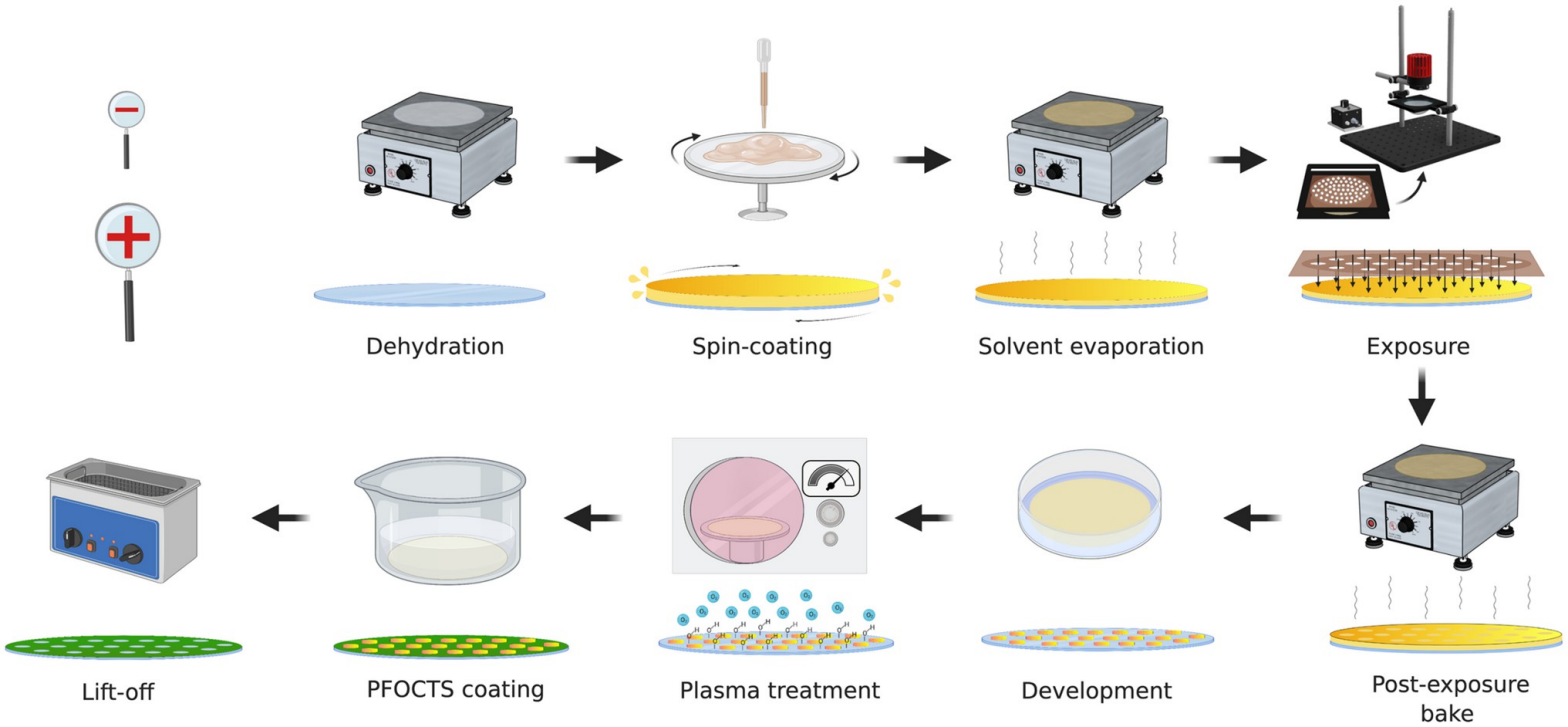
Overview of the fabrication process to produce the hydrophilic-in-hydrophobic spot array outside the cleanroom. Created with BioRender.com.

The MDA substrates were fabricated using 500 μm thick 2” borosilicate glass wafers. The fabrication protocol followed standard UV photolithography procedures applied without the support of CR facilities (Fig 1). The entire process was carried out in a lab with windows covered with a UV filter foil (METOLIGHT SFG10, light cut off: below 520 nm, ASMETEC technical products & service, Germany) to protect the photoresist used for patterning from unintentional exposure to UV light. First, the borosilicate wafer was dehydrated on a hotplate at 250°C for at least 30min. Evaporating the residual water was necessary to avoid possible interferences with the photoresist spin coating step. The wafer was successively cooled down and then placed in a spin coater (SPIN150i-NPP Single Substrate Spin Processor, SPS-Europe B.V., The Netherlands). 2 mL of negative photoresist AZ^®^ nLOF 2020 was pipetted on the centre of the wafer, which was then spin-coated for 50 s at a spin speed of 5000 rpm, with an acceleration of 1000 rpm/s. The centrifugal force spread the resist evenly over the surface. The spin-coated wafer was successively placed on a hotplate for 1 min at 110°C to let the solvent evaporate, as any solvent residual would interfere with the following lithography step. For photopatterning, an in-house built system for collimating light (i.e., for making the light rays parallel to each other) was assembled using a 365 nm LED, an aspheric condenser lens and other supporting components, all purchased from Thorlabs (Thorlabs GmbH, Germany). The system is shown and described in greater details in the next section. The spin-coated wafer was placed on a CO_2_ laser-cut poly(methyl methacrylate) (PMMA) mask holder (**S1 Fig in** S1 File), and covered with a photomask (see **Section 2.4** for a detailed description of the photomask). The holder was then positioned under the aspheric condenser lens and the resist film was exposed to 365 nm light for 30 s with an exposure intensity of approximately 13 mW/cm^2^. During the exposure step, the areas of negative photoresist exposed to light were crosslinked, while the areas shadowed by the photomask remined uncured. The exposed photoresist was then post-exposure baked for 2 min at 110°C to increase its degree of crosslinking and hence to increase the stability of the structures prior to the next development step. Afterwards, the wafer was placed in a shallow beaker with AZ 726 MIF Developer, a tetramethyl-ammonium hydroxide (TMAH)-containing solvent, for 5 min to dissolve most of the non-crosslinked photoresist. Successively, in order to remove any residuals of uncured photoresist from the glass surface and increase its concentration of hydroxyl groups, the wafer was treated with a 15 min oxygen plasma at a power of 990 W. To generate the hydrophobic coating around the hydrophilic spots, the wafer was immersed for 15 min in a 0.4% (v/v) solution of PFOCTS, a fluorinated silane compound, in the fluorinated solvent Novec 7200 contained in a closed beaker inside the fume hood. The wafer was then rinsed with Novec 7200 and dried with a stream of nitrogen gas. To finally lift-off the nLOF protecting the hydrophilic patches from deposition of PFOCTS, the wafer was placed in a shallow beaker containing NMP and sonicated at 60°C for at least 20 min. At the end of the process, an array of hydrophilic glass spots surrounded by a hydrophobic monolayer of PFOCTS was obtained.

### 2.3. Optical setup for photolithography

**Fig 2:**
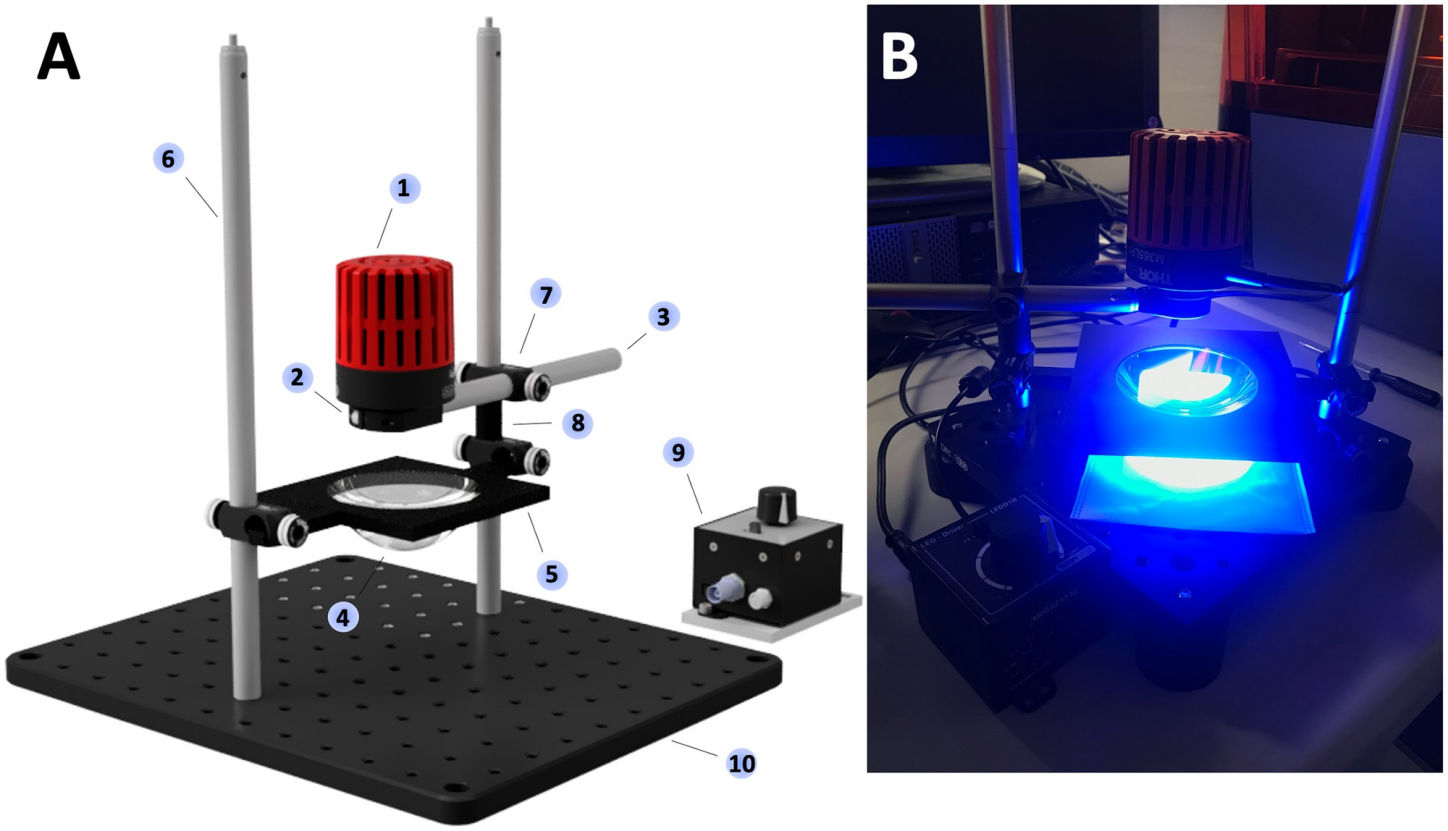
Optical setup assembled to collimate the light and photo-crosslink the resist in the UV photolithography process for fabricating hydrophilic-in-hydrophobic MDAs outside the cleanroom. A) 3D model of the system and B) Photograph displaying the setup with the 365 nm LED powered on. Republished from www.thorslabs.com under a CC BY license, with permission from Thorlabs.

The optical system for collimating the light for the photolithography process (Fig 2) was built using components purchased from Thorlabs GmbH, Germany (the catalogue numbers are given as appropriate and the numbers in brackets refer to the corresponding numbers in Fig 2). Specifically, the setup was composed of a (**1**) 365 nm mounted LED (M365LP1–365 nm, 1350 mW (Min) Mounted LED, 1700 mA) held in place by a (**2**) slip ring (SM1RC/M—Slip Ring for SM1 Lens Tubes and C-Mount Extension Tubes, M4 Tap) connected to an (**3**) optical post (TR150/M—Ø12.7 mm Optical Post, SS, M4 Setscrew, M6 Tap, L = 150 mm). An (**4**) Aspheric Condenser Lens (ACL7560U-A—Aspheric Condenser Lens, Ø75 mm, f = 60 mm, NA = 0.61, ARC: 350–700 nm) for collimating the LED light was placed on a (**5**) black PMMA holder at 27.3 mm from the LED. The lens holder was made of two overlapped 100 mm x 100 mm pieces of black PMMA, where the top one was 3 mm thick and the bottom one was 5 mm thick. The holes fitting the lens, with diameters of 75.1 and 74.8 respectively, were cut out using a CO_2_ laser. The optical post holding the LED and the lens holder were mounted on (**6**) two vertical optical posts (TR300/M—Ø12.7 mm Optical Post, SS, M4 Setscrew, M6 Tap, L = 300 mm) through (**7**) right-angle clamps (RA90/M-P5—Right-Angle Clamp for Ø1/2" Posts, 5 mm Hex, 5 Pack). A (**8**) 3D printed spacer was then placed in between the lens holder and the LED holder to ensure that the correct distance between the lens and LED was maintained. A (**9**) T-Cube LED Driver (LEDD1B - T-Cube LED Driver, 1200 mA Max Drive Current) was used to control the switching on and off of the light source. The entire setup was assembled on a (**10**) Thorlabs standard aluminium optical breadboard.

Taking into account that with such a basic optical system the output beam could not be perfectly collimated, a distance of 27.3 mm between the LED and the lens was chosen, based on the observation of the light beam image on a white piece of paper. It looked overall homogenous and, moving the LED and the lens away from the piece of paper, no changes were observed in the beam diameter over a range of about 20 cm in hight from the breadboard. With the described setup, an energy intensity of approximately 13 mW/cm^2^ was measured with a power meter (Thorlabs GmbH, Germany) in the centre of the collimated light beam when the lens was placed 10 mm above the wafer surface.

### 2.4. Pattern characterization

The usability of the photolithography process for fabricating hydrophilic-in-hydrophobic MDAs was investigated by patterning the photoresist with a photomask (5inch Soda Lime mask CD 1.0 micron, Compugraphics International Ltd., UK) containing 32 spot arrays with varying spot diameters and pitches. The photomask was designed using the layout editor software CleWin 5 (WieWeb software, The Netherlands). The arrays were composed of spots with diameters of 2, 4 and 6 μm with edge-to-edge pitches of 15, 25, 50, and 75 μm respectively, spots with diameters of 8, 10 and 12 μm with edge-to-edge pitches of 25, 50, 75, and 100 μm respectively, and spots with diameters of 16 and 20 μm with edge-to-edge pitches of 50, 75, 100 and 150 μm respectively. However, only the arrays with spot diameters varying between 4 and 16 μm were tested because these spot sizes were suitable for fabricating substrates for MDA-based assays. Brightfield images of the resist patterns were taken on a Primovert inverted widefield microscope (ZeissTM, Germany) and on a AxioObserver Z1 (ZeissTM, Germany) equipped with a high-resolution camera (Axiocam 712mono, Zeiss^™^, Germany).

### 2.5. Surface functionalisation

The patterned wafer was functionalized with epoxy groups through a silanization process. Before silanizing the hydrophilic spot surfaces, the wafer was washed for 5 min in acetone, isopropanol and ethanol, respectively, to remove any dust particles or debris from the fabrication process. Next, the wafer was immersed in a 1% (v/v) solution of GLYMO in 96% (v/v) ethanol for 2 h at room temperature. After being removed from the solution, the wafer was placed on a hotplate at 110°C for 1 hour for curing, rinsed in ethanol, and finally dried with a stream of nitrogen gas. Shortly after silanization, solutions of the proteins used for testing the fabricated substrates were deposited on the patterned surface and incubated under a coverslip overnight at 4°C. The covalent attachment was induced by the reaction between the amino groups on the protein surface and the epoxy group of the silane. Verification of the surface functionalization process was done by immobilizing Anti-CD63 antibody, previously conjugated with FITC according to the manufacturer’s instructions. For this purpose, a 6 μM solution of the green-fluorescent antibody was prepared in 0.01 M phosphate-buffered saline (PBS) (pH 7.4) containing 0.1% (v/v) of Tween-20. After incubation, the wafer was rinsed in PBS buffer and visualized in a microscope.

### 2.6. Microdroplet array generation

**Fig 3:**
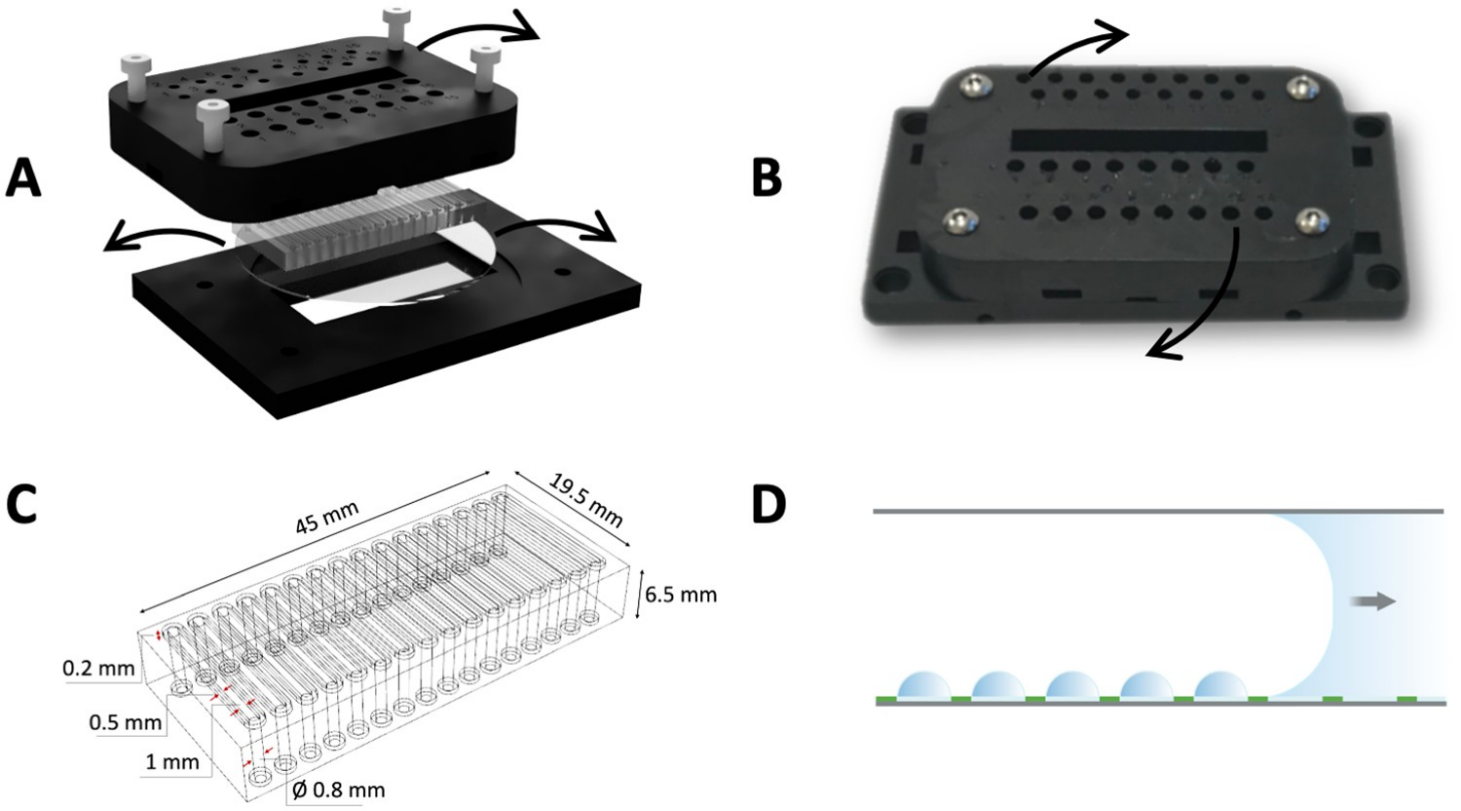
A) Expanded microfluidic device composed of a clamping system printed in Formlabs black resin and a PDMS gaskets that interfaces with the patterned 2” glass wafer forming micro-channels when the device is assembled. B) Picture of the assembled microfluidic device indicating the inlets, used for loading the liquid reagents inside the channels, and the outlets, used to connect with a peristaltic pump. C) Schematic of the PDMS gasket that forms 16 identical channels when pressed on top of the glass surface, with inlets and outlets coinciding with the ones in the 3D printed holder. The channels are 1 mm wide, 15 mm long and 100 μm high. D) Illustration of the mechanism for droplet formation: the hydrophilic glass circles, which are surrounded by a hydrophobic coating (green), retain some of the liquid when a water plug is pumped over the patterned surface through the use of the PDMS micro-channels.

The patterned wafer was assembled into a microfluidic device composed of a 3D printed holder and a PDMS gasket forming 16 identical channels (1 mm wide, 15 mm long and 100 μm high) when interfaced with the glass surface (Fig 3A–3C). The holder contained inlets and outlets, the latter connected to a peristaltic pump that was used to introduce liquids inside the channels by applying a negative pressure. The MDAs were generated by passing a water plug over the substrate surface (Fig 3D). In this way, the water was retained in the hydrophilic spots but removed from the hydrophobic background, leading to the quick and spontaneous formation of tens of thousands of femtoliter-sized droplets with a water/air interface.

### 2.7. Single molecule counting on HiH-MDAs

Total Tau protein (100nM solution in 0.01 M PBS, pH 7.4) was incubated on a GLYMO-functionalised substrate for 1 h and subsequently washed 5 times with PBS. A dilution series of anti-Tau antibody conjugated with HRP (100 fM, 1fM, 100aM, 10aM) and a negative control were prepared in the assay buffer (10 mM PBS, 136 mM NaCl, 1% (w/v) BSA, 0.1% (v/v) Tween-20, pH 7.4). BSA was here used as blocking agent to lower the unspecific binding of the analyte to the surface. Each of the antibody solutions was loaded in a separate channel, making three replicates per concentration, and left incubating for 2 h at room temperature to allow for the antibodies to reach the surface and interact with their antigen. The channels were then washed 5 times with PBS buffer to remove the unbound molecules. Lastly, a fluorogenic HRP-substrate solution (18 mM H_2_O_2_ and 200 μM AFR in PBS buffer with 0.1% (v/v) Tween-20) was prepared, and 10 μL was pumped through the channel to produce the droplet array. After a few seconds of incubation, a strong fluorescent signal was generated inside the droplets containing the labelled analyte due to the enzyme-catalysed oxidation of the AFR. The number of signal-emitting droplets was digitally counted using a fluorescence microscope and correlated to the known antibody concentrations to generate a calibration curve.

### 2.8. Image acquisition and data analysis

All fluorescence images were acquired on an Axio Observer Z1 fluorescence microscope (ZeissTM, Germany) equipped with a high-resolution camera (Axiocam 712mono, ZeissTM, Germany) and Zeiss Zen imaging software. AFR product was imaged using Zeiss filter set 43HE (EX550/25, EM605/70), Zeiss Colibri.2 555 nm LED and Zeiss EC Plan-Neofluar 10x/0.30 Ph1 objective. FITC was imaged using Zeiss filter set 38HE (EX470/40, EM525/50), Zeiss Colibri.2 480 nm LED and Zeiss LD Plan-Neofluar 20x/0.4 Korr M27 objective. All the fluorescence micrographs were processed for display using Fiji [22]. For digital counting, the fluorescence signals were binarized using consistent intensity threshold values, that were kept the same for all the fluorescence micrographs from the same experiment.

## 3. Results and discussion

### 3.1. nLOF-based process for fabricating MDAs

**Fig 4:**
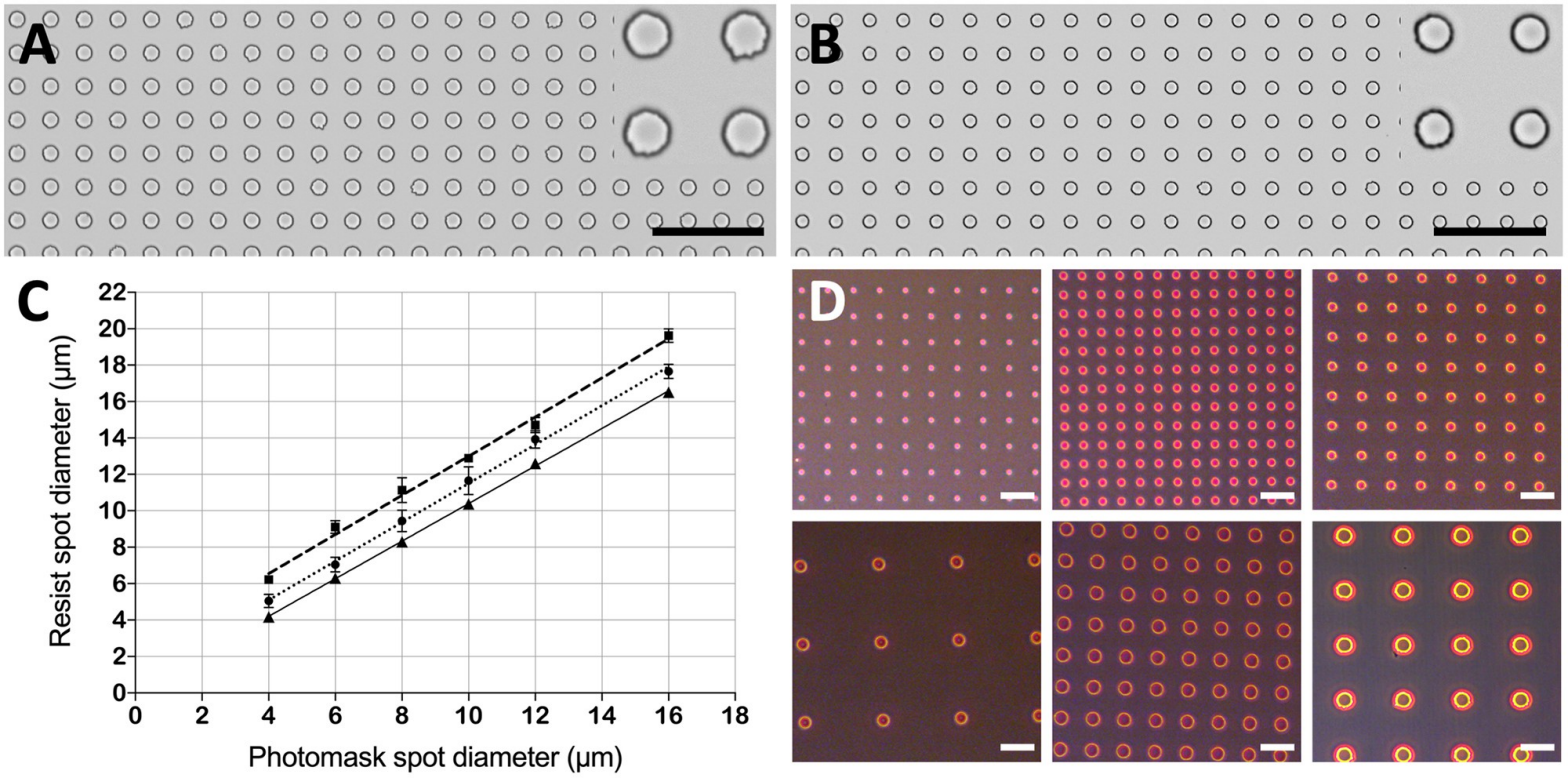
A, B) Brightfield micrographs displaying nLOF resist spot arrays with 10 μm spot diameter and 20 μm pitch produced in soft contact mode (A) and hard contact mode (B), respectively. Scale bars are 100 μm. C) Linear regressions showing the correlation between the photomask feature sizes and the dimensions obtained in soft contact (squares, dashed line, R^2^ = 0.994, Y = 1.075*X + 2.25), hard contact (circles, dotted line, R^2^ = 0.998, Y = 1.068*X + 0.8311), hard contact with the CR-based nLOF process (triangles, full line, R^2^ = 0.999, Y = 1.032*X + 0.07767). The error bars represent standard deviations of 10 repetitions. D) Brightfield micrographs displaying representative nLOF resist spot arrays with varying diameters and pitches (i.e., top left 4 μm diameter and 25 μm pitch, top middle 6 μm diameter and 15 μm pitch, top right 8 μm diameter and 25 μm pitch, bottom left 10 μm diameter and 75 μm pitch, bottom middle 12 μm diameter and 25 μm pitch and bottom right 16 μm diameter and 50 μm pitch, respectively) reproduced with exposure in soft contact mode. Scale bars are 40 μm. Note that the brightfield micrographs in A-B and D are taken with different microscopes.

The CR fabrication process previously developed in the group was based on the molecular vapor deposition (MVD) of perfluorodecyltrichloro-silane (FDTS) where the hydrophilic spots were protected by the positive photoresist AZ 5214E (the general procedure is described in **S2 Fig in** S1 File) [21]. The main issue with this process was its dependency on equipment, such as the MVD machine, shared among all the CR users. In particular, poor adhesion of the FDTS layer was periodically experienced, which was likely due to impurities from other processes despite rigorous cleaning of the MVD chamber prior to use. The unstable hydrophobic layer often prevented the MDA generation and hence resulted in failed experiments (**S3 Fig in** S1 File). To overcome this problem, we sought for an alternative method, involving the formation of the hydrophobic layer in a solution freshly prepared each time. For this purpose, a negative photoresist (nLOF) was tested as protective layer during the liquid-phase silanization with trichloro(1H,1H,2H,2H-perfluorooctyl)-silane (PFOCTS), since a positive photoresist cannot withstand the necessary solvents for a wet chemistry process. PFOCTS is a fluorinated compound that has comparable hydrophobic properties as FDTS. The photolithography procedure was carried out using CR standard equipment, while the silanization and nLOF removal were performed outside the CR, in chemically controlled condition, to evaluate the impact of the chemistry involved (**S4 Fig in** S1 File). The resulting resist spot size was on average 0.3 μm larger than the actual feature dimension in the photomask. The spot-to-spot coefficient of variation (CV%) was less than 2% over a broad range of spot sizes (Fig 4C, **triangles**).

The generation of the hydrophobic coating in solution using PFOCTS resulted in a more stable monolayer compared to the FDTS coating generated using the shared molecular vapor deposition (MVD) machine in the CR. In fact, with this new process, peeling of the hydrophobic layer as often obtained with the previous MVD-based method (see **S3 Fig in** S1 File) was not observed. The standard MVD operating procedure is based on the use of a vacuum chamber where the wafer to be coated is placed. Inside the vacuum chamber, in the presence of an oxygen plasma, a vaporised fluorinated silane compound, such as FDTS, reacts with the glass surface generating a monolayer-thick fluorocarbon film, without the need of a solvent. By contrast, the new process for coating the wafers was based on immersing the patterned wafer into a diluted solution of PFOCTS which, even if very volatile, remains mostly in its liquid form at a standard atmospheric pressure, enabling liquid-phase silanization of the glass. With this method, the fluorinated silane compound was in close contact with the entire patterned wafer and formed siloxane bonds with the glass surface not covered by the nLOF [23]. The PFOCTS deposited on top of the nLOF spots was then removed together with the photoresist after the final lift-off step, which exposed the glass patches that should remain hydrophilic at the end of the process. This method resulted in reproducible generation of MDAs over the patterned surface when the glass substrate was assembled into the 3D printed microfluidic system and an aqueous solution (PBS) was pumped across the surface. Additionally, the contact angles of the hydrophobic and hydrophilic areas measured 120° and 40°, respectively, in agreement with the values reported for the previously published protocol [21], for which the nLOF-based process was therefore considered as a good replacement. The high contact angles indicated that the covalently glass linked PFOCTS was functionally unaffected by the removal of nLOF structures.

### 3.2. CR-free nLOF-based process: Pattern characterization

The photolithography parameters employed for the nLOF-based CR procedure were successively adjusted to pattern the substrates outside the CR using an in-house built small collimated light system. For resist photopatterning, two exposure modes were tested: soft contact and hard contact. The soft contact was realized by simply placing the photomask on top of the wafer by using a holder that was produced in black PMMA to avoid light reflections. For the hard contact, instead, the photomask was clamped on top of the substrate by applying pressure through the use of two aluminium bars on the two mask sides, kept in place by a set of 4 M6 x 25 mm knurled thumb screws and M6 wing nuts. The exposure time was set manually using a digital timer. However, with a duration of 30 s to ensure a complete resist crosslinking, minor variations in the exposure time were considered acceptable. Additionally, as the MDA is a repetitive, non-directional, pattern, precise alignment in X/Y was not necessary.

The soft contact mode enabled a very quick substrate fabrication but produced resist spots that were 2 to 3 μm larger than the corresponding photomask feature sizes due to diffraction effects at the edges of the exposed patches (Fig 4A and 4C, **squares**). By contrast, the hard contact mode produced resist spots that were on average only 1.1 μm larger in diameter compared to the CR-based process, thus resulting in good agreement between obtained dimensions and photomask dimensions (Fig 4B and 4C, **circles**). The spot-to-spot size CV% across the entire substrate was about 3% for soft contact mode and 5% for hard contact mode, respectively, with high batch-to-batch reproducibility. A deeper analysis of the spot sizes showed that for the soft contact mode the spot-to-spot size CVs% in the centre, halfway out and at the edge of the wafer were 3.0%, 2.8% and 3.3% respectively, and that there was no statistically significant variation in spot size from the centre to the edge. Therefore, as these CV% values were very close to the value obtained for the CR-based process (*i*.*e*., 2%), the home-built collimated light source provided sufficient consistency for the MDA substrates fabrication. The larger CV% obtained for the hard contact could be explained by the method used for clamping the photomask on top of the wafer. It is possible that such imprecise method resulted in uneven pressure exerted on the mask surface, causing larger variation of the spot sizes, which could be improved by using a more refined clamping system. It was not possible to measure the distance between the photomasks and the substrate to confirm this or to correlate with the larger spots of the soft contact mode. The soft contact mode resulted in more spots with rough edges compared to the hard contact (Fig 4A and 4B, **insets**). However, the linear relationship between the photomask feature dimensions and the obtained dimensions indicated a good pattern transfer also using the soft contact mode (Fig 4C and 4D), and hence its feasibility for producing MDA substrates.

**Fig 5:**
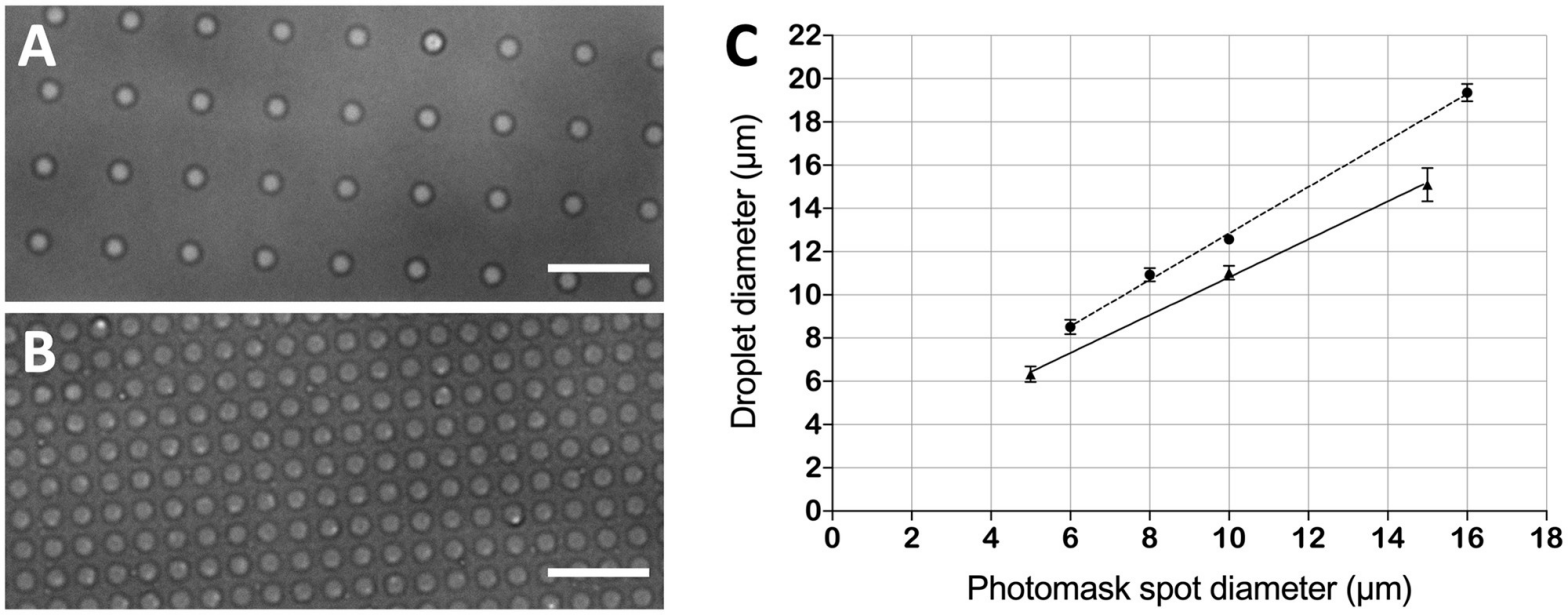
A) Brightfield micrograph displaying a microdroplet array with 10 μm spot diameter and 25 μm pitch produced in soft contact mode. (B) Brightfield micrograph displaying a microdroplet array with 10 μm spot diameter and 5 μm pitch produced in hard contact mode with the previously published CR-based method [21]. Scale bars are 50 μm. C) Linear regressions showing the correlation between the photomask feature sizes and the droplet sizes obtained by producing the MDA substrates in soft contact with the nLOF-based CR-free method (dashed line, R^2^ = 0.997, Y = 1.075*X + 2.092), and in hard contact with the previously published CR-based process (full line, R^2^ = 0.998, Y = 0.877*X + 2.047). The error bars represent standard deviations of 10 repetitions. Droplets on 4 μm diameter spots are not available.

Consequently, the soft contact was chosen as exposure mode for photopatterning in all the subsequent experiments, since it enabled a much faster fabrication of the MDA substrates compared to the hard contact. Despite the slightly larger dimensions of the droplets obtained with the described fabrication strategy in soft contact compared to the previously published CR-based protocol [21], high-quality MDAs were produced (Fig 5A **and S5 Fig in** S1 File). The increase in droplet dimension could be compensated with smaller features in the photomask, if necessary. The rough edges of the resist spots did not influence the droplet shape, which showed a droplet-to-droplet size variation of only 2.6%.

As the actual MDA spot size was less important than the uniformity of the dimensions, we also tested a non-collimated light source (i.e., a UV flood light) in hard contact mode, which would further simplify the method by exploiting instruments that are commonly used in many labs. However, only one of the arrays tested with various spot sizes and pitches gave consistent spots, but with a diameter about 7-8-fold larger than expected (**S6 Fig in** S1 File). This result showed the need for collimated light in achieving well-developed photo-patterns.

In order to demonstrate that the hydrophilic spots consisted of pure glass, an MDA substrate produced with the CR-free process was first functionalized with epoxides and, subsequently, FITC-labelled Anti-CD63 antibodies were covalently bound to the surface. Fluorescence microscopy showed that the fluorescently labelled antibodies were specifically immobilized on the hydrophilic patches, as no fluorescence was detected on the hydrophobic surrounding (**S7 Fig in** S1 File).

### 3.3. Single-molecule counting on HiH-MDAs with water/air interface

**Fig 6:**
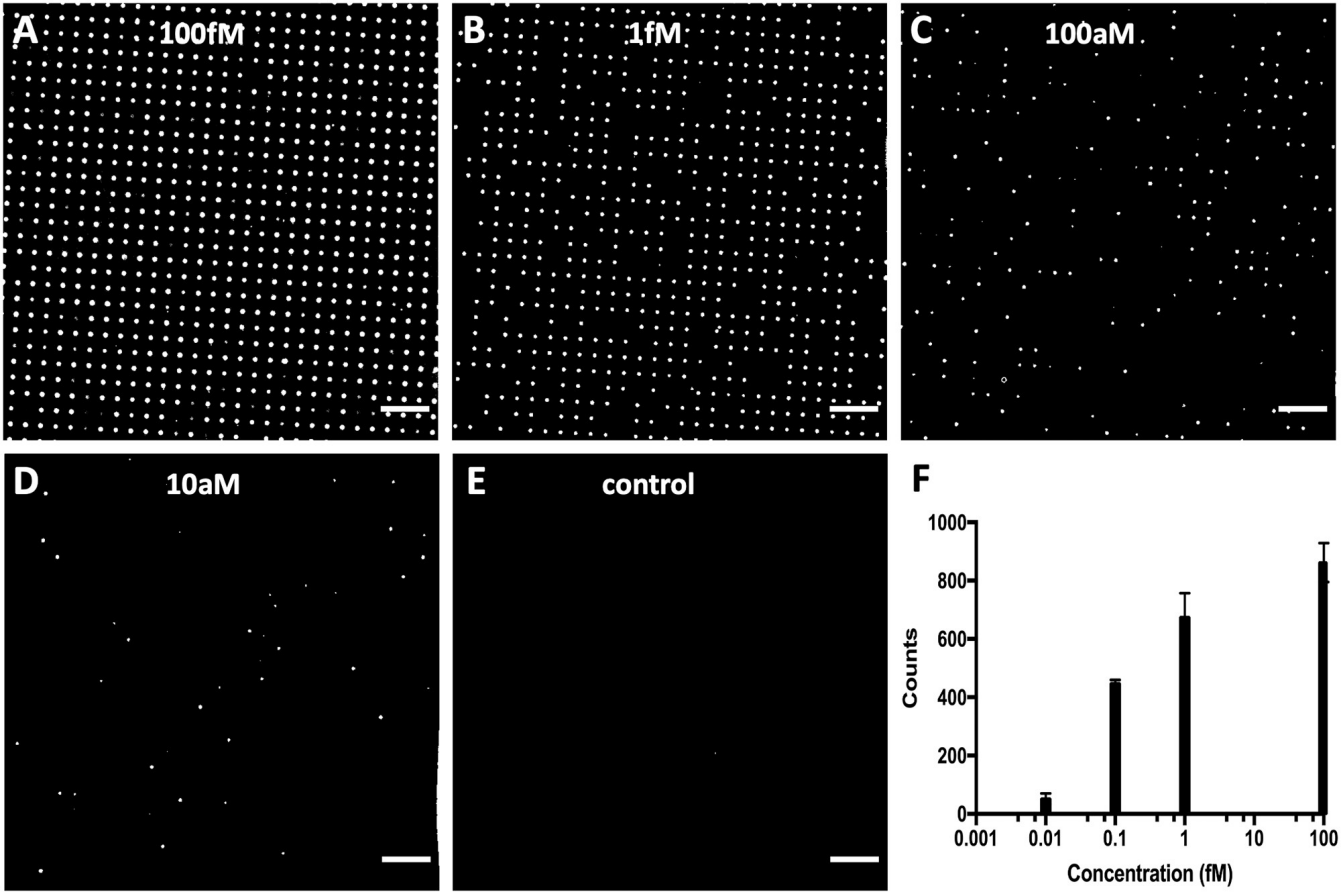
Hydrophilic-in-hydrophobic MDA applied to enzyme linked immunosorbent assay for single molecule counting. Representative fluorescence micrographs of the dilution series obtained with different concentrations of Anti-Tau antibody conjugated with HRP: A) 100 fM, B) 1 fM, C) 100 aM, D) 10a M and E) control. Scale bars are 100μm. F) Bar graph generated from the quantification of the number of spots in the MDA in a frame (890 μm x 1035 μm) imaged with the 10x objective for each concentration (n = 3).

As a use case, a simplified version of digital ELISA was performed to evaluate whether the MDA substrates fabricated outside the CR produced by soft contact mode were suitable for detecting analytes with a single-molecule counting method. The digital ELISA technique is commonly used for the analysis of samples containing extremely low concentrated analytes, typically in the femto- and attomolar range [11]. The analytes are immobilized using specific capture probes covalently bound to the surface of the tens of thousands of hydrophilic spots composing the array, and then labelled with a reporter enzyme. Successively, a fluorogenic enzyme substrate solution is used to generate the MDA, where each droplet statistically contains only zero or one analyte molecule. Therefore, after a few seconds of incubation, a strong fluorescent signal is developed only inside the droplets containing the single enzyme-labelled target molecules. The number of fluorescent droplets can then be counted and directly correlated with the total number of immobilized molecules, and hence with their concentration in the samples. Specifically, for the described assay, Tau protein was immobilised on the spot surfaces as capture probe and anti-Tau antibody conjugated with HRP was used as analyte. Fig 6 shows representative fluorescence micrographs of a dilution series of anti-Tau-HRP, where the number of fluorescent droplets decreased with the concentration of the target antibody as expected. No fluorescent spots were observed in the control channel corresponding to zero molecules of the antibody, indicating that there is no unspecific fluorescence signal deriving from the substrate that can interfere with the assay outcome. Additionally, the integrity of the MDA was always confirmed through brightfield imaging prior to perform the fluorescence analysis, and no fouling of the surface was visible at the end of the experiments, even in the presence of BSA in the assay buffer. This demonstrated the robustness and reliability of the fabrication method. Therefore, together with the surface chemistry applied, such MDAs are suitable for implementing digital ELISA, where alternated bulk incubations and micro-compartmentalized reactions are involved.

In general, MDAs have been widely used for single molecule counting applications, but the fabrication of the MDA substrates has required CR-related expertise and access to these facilities [1, 19–21, 24, 25]. The new fabrication method removed this obstacle, rendering the MDA technology, and hence many MDA-based assay techniques, accessible to more labs. In fact, in addition to the digital ELISA method described here, there are several other applications that could be performed on the MDA platform, such as the loop-mediated isothermal amplification (LAMP) for the detection and quantification of DNA [26, 27]. LAMP could be easily performed on the described MDA device because it is a technique based on isothermal nucleic acid amplification which, in contrast to polymerase chain reaction methods, does not require sophisticated instrumentation like the thermal cycler for alternating temperature steps. Also other digital assay formats such as digital proximity ligation assays [4] or even single cell-[25] or single exosome-[7] based assays could be implemented, as well as the expression of protein libraries [21], which in the future could be used for epitope mapping.

## 5. Conclusions

As Dittel and Bürkle noted, “… to integrate a complete process in the cleanroom and operate it, represents technically the most difficult and expensive application engineering alternative” [28]. Here we demonstrated that for the given application, a small and around $1000 collimated light source provides sufficient accuracy and precision for photopatterning at the micron-scale. This allowed the development of a robust fabrication process to produce MDA substrates outside the CR that relied on well controlled post-patterning chemical conditions. The obtained MDAs showed characteristics comparable to the devices fabricated following the previously published CR-procedure and an improved stability of the hydrophobic coating. In conclusion, the small instrumentation requirements and the rapid and robust nature of the described process enables the fabrication of HiH-MDAs in a cost-effective manner, independently from the availability of CR facilities. This simplified protocol would hence lower the threshold of using the MDA technology for various bioassays. However, the use of highly toxic chemicals should possibly be replaced with milder ones. For instance, dimethyl sulfoxide (DMSO) could be used as an alternative to NMP for the lift-off process and it would be worth to investigate the use of sodium hydroxide (NaOH) or potassium hydroxide (KOH)-based developers to replace the extremely toxic TMAH developer and render such manual procedure safer for the device manufacturer.

## Supporting information

S1 File The following are available online at www.mdpi.com/xxx/s1, S1 Fig: Optical setup assembled to photo-crosslink the resist in the UV photolithography process for fabricating hydrophilic-in-hydrophobic MDAs outside the cleanroom. S2 Fig: Photomask and wafer holder for photopatterning. S3 Fig: Overview of the fabrication process previously published to produce the hydrophilic-in-hydrophobic spot array inside the cleanroom. S4 Fig: Brightfield micrographs displaying the nLOF 2020 resist spot array and the corresponding MDA, respectively, following the process inside the CR. S5 Fig: Brightfield micrographs displaying arrays of aqueous microdroplets with different sizes and pitches, generated on MDA substrates produced in soft contact with the nLOF CR-free process. S6 Fig: Representative brightfield micrographs of the nLOF resist patterns made by using a not collimated light source. S7 Fig: Fluorescence micrograph showing the selective binding of fluorescently labelled Anti-CD63 antibody on the glass of a patterned wafer.
(DOCX)

S1 Data (XLSX)
