# Supplementary material for: Accessible, fast and easy fabrication of hydrophilic-in-hydrophobic microdroplet arrays
Source: PLoS One. 2022 Feb 25;17(2):e0263282. doi: 10.1371/journal.pone.0263282 (PMC8880433; doi:10.1371/journal.pone.0263282)
Supplement: S1 File — The following are available online at www.mdpi.com/xxx/s1, S1 Fig: Optical setup assembled to photo-crosslink the resist in the UV photolithography process for fabricating hydrophilic-in-hydrophobic MDAs outside the cleanroom. S2 Fig: Photomask and wafer holder for photopatterning. S3 Fig: Overview of the fabrication process previously published to produce the hydrophilic-in-hydrophobic spot array inside the cleanroom. S4 Fig: Brightfield micrographs displaying the nLOF 2020 resist spot array and the corresponding MDA, respectively, following the process inside the CR. S5 Fig: Brightfield micrographs displaying arrays of aqueous microdroplets with different sizes and pitches, generated on MDA substrates produced in soft contact with the nLOF CR-free process. S6 Fig: Representative brightfield micrographs of the nLOF resist patterns made by using a not collimated light source. S7 Fig: Fluorescence micrograph showing the selective binding of fluorescently labelled Anti-CD63 antibody on the glass of a patterned wafer. (DOCX) [file pone.0263282.s001.docx]

Supplementary Materials

Accessible, fast and easy fabrication of hydrophilic-in-hydrophobic microdroplet arrays

Arianna Toppi, Martin Dufva *

| 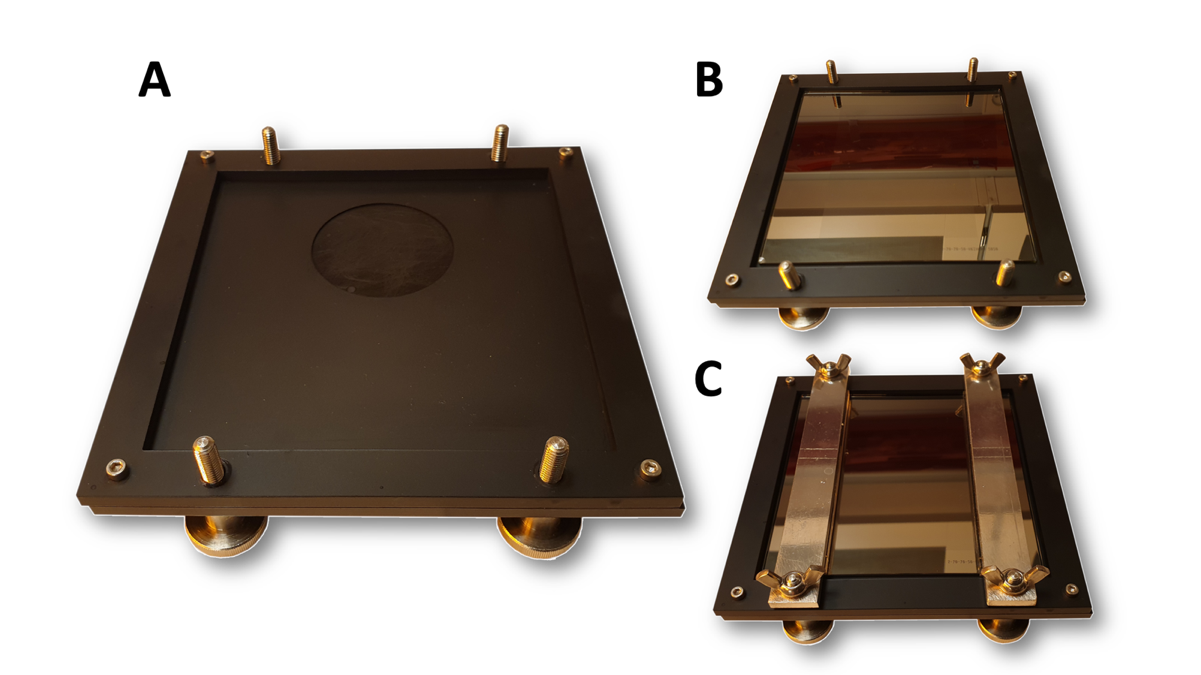 |
| --- |
| **Figure S1.** Photomask and wafer holder for photopatterning. A) PMMA CO_2_ laser cut holder with the recess for placing a 2” wafer. B) Photomask placed on top the wafer for soft contact exposure and C) photomask clamped on top of the wafer for hard contact exposure. |

| 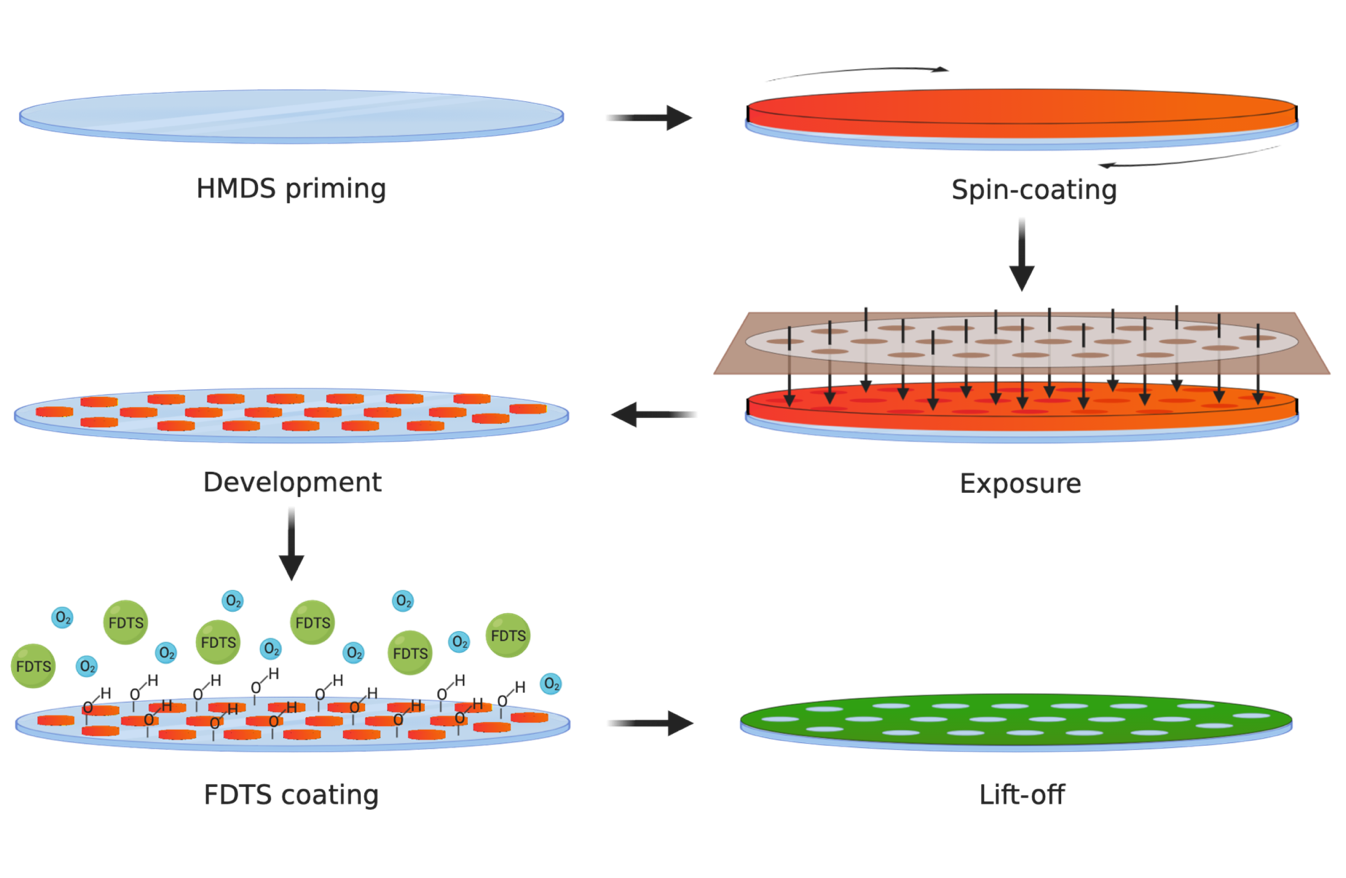 |
| --- |
| **Figure S2.** Overview of the fabrication process previously published(1) to produce the hydrophilic-in-hydrophobic spot array inside the cleanroom using a positive photoresist and a monolayer of FDTS deposited from a gas phase as hydrophobic coating. Created with BioRender.com |

| 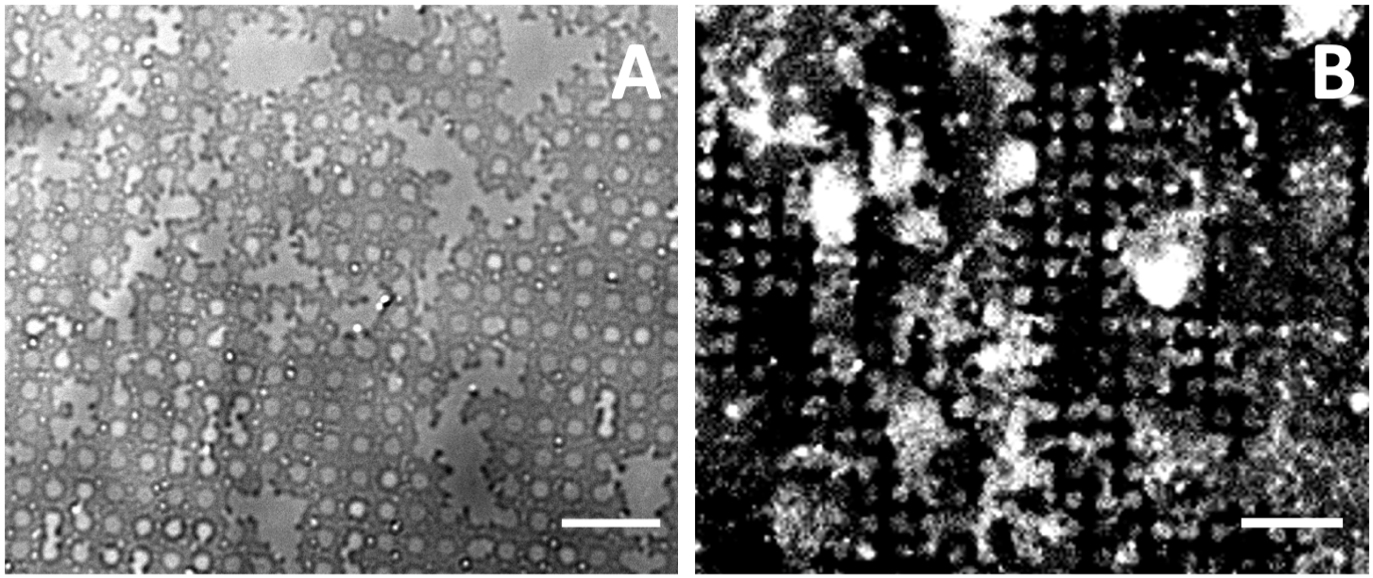 |
| --- |
| **Figure S3.** A) Brightfield and B) fluorescence micrographs displaying an example of MDA generated on a FDTS-based substrate fabricated in the CR and damaged after performing a digital ELISA assay. Scale bar is 50 µm. |

| 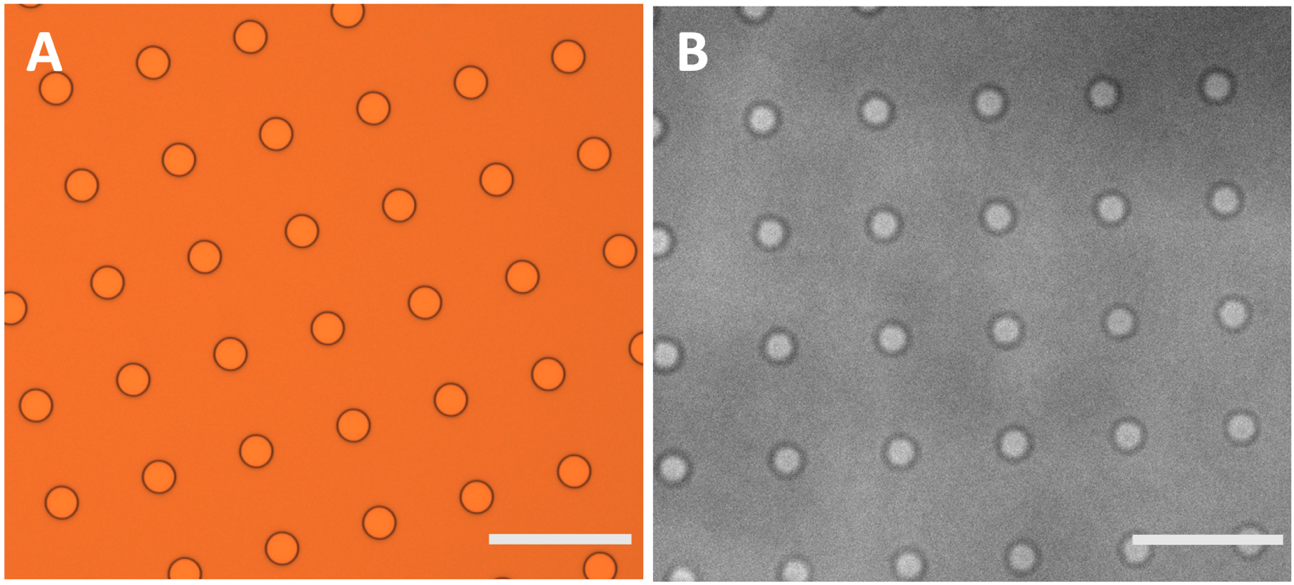 |
| --- |
| **Figure S4.** A) Representative brightfield micrograph displaying the nLOF 2020 resist spot array with 12 μm spot diameters and 25 μm pitches, reproduced with exposure in hard contact mode following the process inside the CR. The obtained spot diameter was 12.5 ± 0.1 µm. B) Brightfield micrograph of the corresponding microdroplet array generated on the patterned substrate after formation of the PFOCTS hydrophobic coating in solution and removal of the resist, outside the CR. Scale bars are 50 μm. |

| 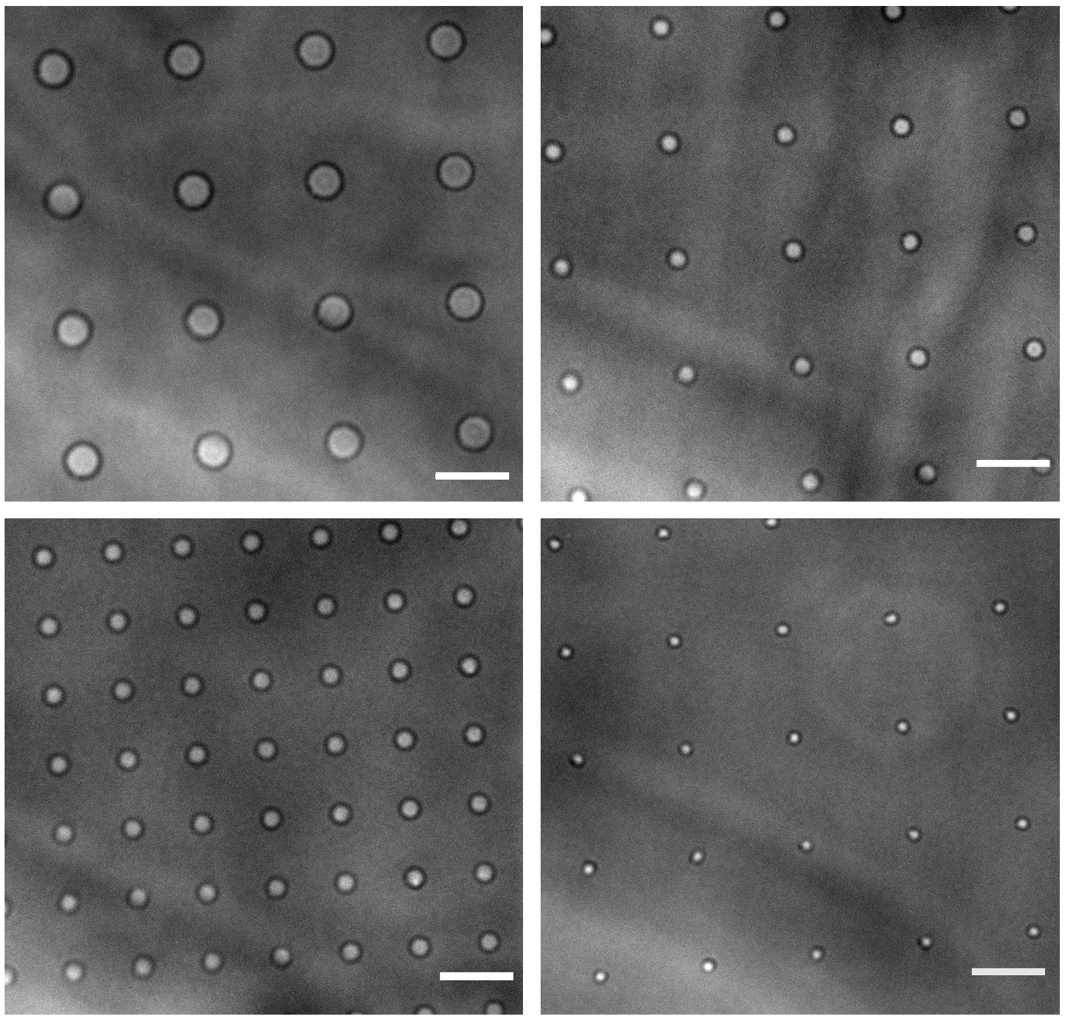 |
| --- |
| **Figure S5.** Brightfield micrographs displaying arrays of aqueous microdroplets with different sizes and pitches, generated on MDA substrates produced in soft contact with the nLOF CR-free process. The hydrophilic spot diameter varies between 16 μm (top left) and 6 μm (bottom right). Scale bars are 40 μm. |

*Effect of non-collimated light on photopatterning*

The use of UV flood lamp (Dymax 5000-EC UV flood lamp, Dymax corporation, USA) for exposing the photoresist was tested to check if a non-collimated light source could be used for photopatterning. As expected, the result obtained using the same photomask with the 32 arrays of circles with varying diameters and pitches showed that only the larger features could be transferred (See Figure S6, SI “16_50”, where 16 indicates the spot diameter and 50 the edge-to-edge pitch), while the rest of the patterns were far from the desired spot morphology possibly due to the effect of light diffraction. However, also in the case of larger features such as 16 μm diameters and 50 μm spacing, even if likely usable, the structures were much larger than expected, which makes non-collimated light sources difficult to use.

| 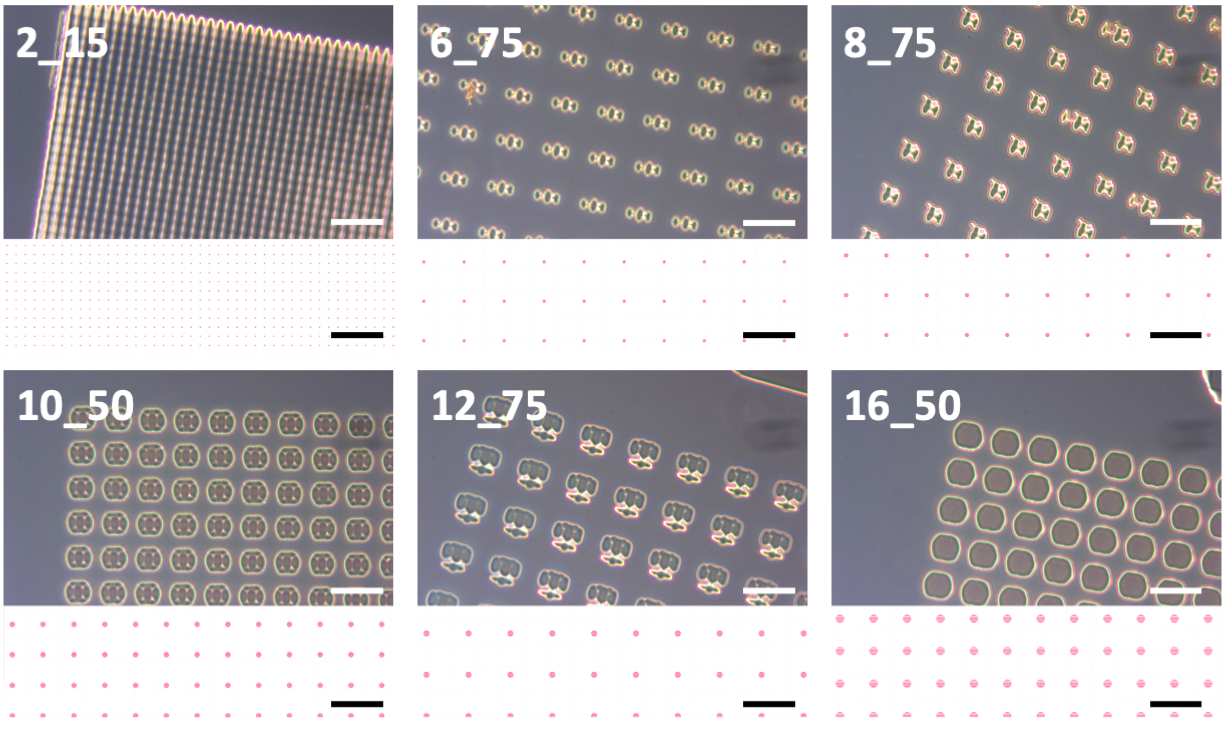 |
| --- |
| **Figure S6.** Representative brightfield micrographs of the nLOF resist patterns made by using a non-collimated light source (top) and the corresponding patterns in the original photomask (bottom). The numbers in the top left corners of the micrographs represent the spot diameter and edge-to-edge pitch, respectively. Scale bars are 100 μm. For the exposure was used a Dymax 5000-EC UV flood lamp with an intensity in the UVA range (320-390 nm) of 225 mW/cm^2^. Exposure time: 45 s. |

| 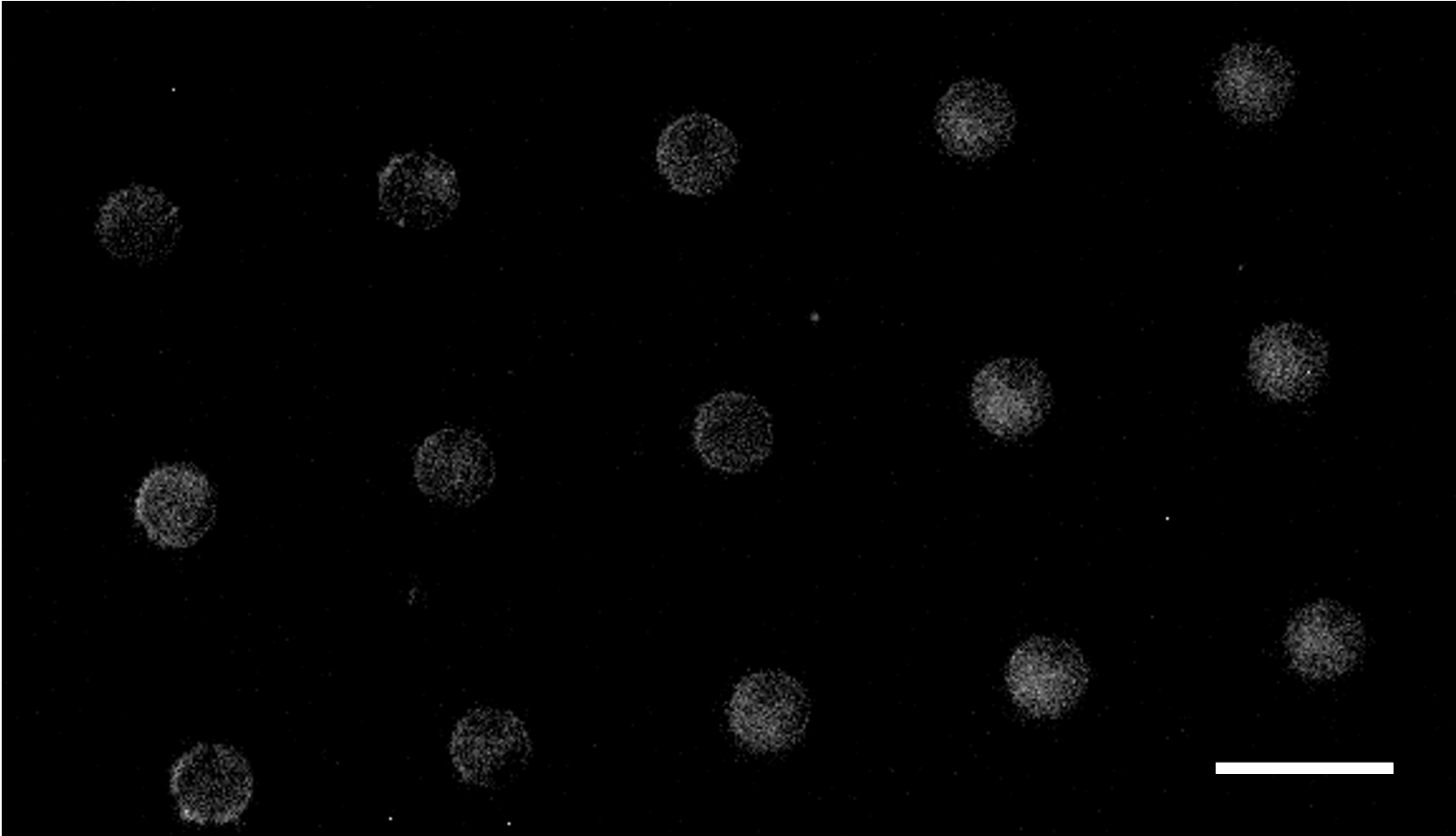 |
| --- |
| **Figure S7.** Fluorescence micrograph showing the selective binding of fluorescently labelled Anti-CD63 antibody on the glass of a patterned wafer. Scale bar is 50 μm. |

1. Kunding AH, Busk LL, Webb H, Klafki HW, Otto M, Kutter JP, et al. Micro-droplet arrays for micro-compartmentalization using an air/water interface. Lab Chip. 2018 Sep 21;18(18):2797–805.
